# Giant Omphalocele Complicated by Postoperative Duodenal Obstruction

**DOI:** 10.21699/ajcr.v8i1.518

**Published:** 2017-01-05

**Authors:** Sunita Ojha, Shobha Parashar, Dharmil Doshi, Rajiv Kumar Bansal

**Affiliations:** 1 Department of Pediatric Surgery, Santokba Durlabhji Memorial Hospital and Research Institute, Jaipur; 2 Department of Anesthesiology, Santokba Durlabhji Memorial Hospital and Research Institute, Jaipur; 3 Department of Pediatrics, Santokba Durlabhji Memorial Hospital and Research Institute, Jaipur

**Keywords:** Giant omphalocele, Duodenal obstruction, Postoperative complication

## Abstract

Omphalocele is a congenital defect in the abdominal wall, usually treated at birth or within 1-2 years of life depending on condition of patient and size and contents of the defect. We repaired a giant omphalocele without mesh in a 9-year-old girl. She developed duodenal obstruction in the postoperative period requiring another laparotomy and duodeno-jejunostomy to bypass obstruction.

## CASE REPORT

A-9-year-old girl presented to us with giant omphalocele (ventral hernia). Primary repair at birth was attempted but could not be achieved because of huge size and protruding liver. Gradually, the lump increased in size and concern of the lump being a tumor forced the parents to seek a medical advice at 9 year of age. Child initially had no problems but for the last 2-3 years she had vomiting on straining. On examination, there was a large lump (12cm x 14cm) in the anterior abdominal wall extending from the xiphisternum to the level of iliac crest (Fig.1) This lump was firm and barely could be compressed down into the abdomen. Remaining systemic examination was essentially normal. Abdominal skiagram showed a radiopaque shadow in the mid abdomen, and bowel loops all around it. Normal Liver shadow was not seen below the right diaphragm; rather it was occupied by the bowel loops. Ultrasonography revealed the right lobe of liver with gall bladder lying anteriorly to the left lobe and bulging into the omphalocele. On Doppler scan, inferior vena cava and portal vein were also displaced and anteriorly placed. Rest of the structures were normal.

**Figure:**
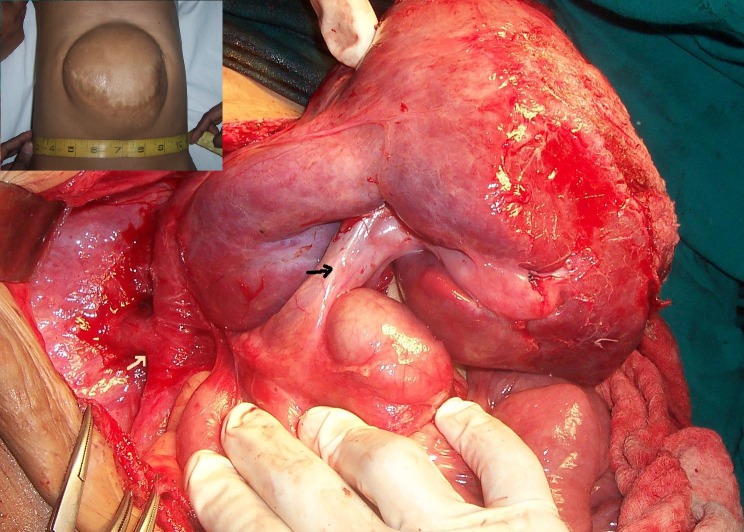
Figure 1: Intraoperative photograph showing complete right lobe of liver protruding out of the abdominal wall. White arrow showing the IVC, black arrow at the CBD. Inset shows preoperative picture.

At operation, skin was densely adherent to the liver surface. Complete right lobe of liver with gall bladder, distal part of stomach and first part of duodenum were lying anteriorly in the omphalocele. Portal vein was quite anterior and medial in position. After separation of adhesions whole of right lobe of liver was lying outside the abdominal cavity and IVC and hepatic veins were visualized (Fig.1). After abdominal wall stretching the liver was put below the right hemidiaphragm. Rectus muscles were mobilized and primary closure was done although under tension. While closing the abdomen blood pressure, CVP, and vitals were strictly monitored. Urinary bladder pressure preoperatively was 4 cm of H2O, after the closure it was 12 cm of H2O. Postoperatively child was managed on high intravenous fluid to take care of increased abdominal pressure for about 24 hours. Postoperatively, the child remained well hemodynamically, but significant NG tube aspirates persisted for 9 days. Contrast study revealed distended stomach and very slow transit beyond the first part of duodenum. Upper gastrointestinal endoscopy revealed dilatation of the first part of duodenum (D1) with kinking and inability to negotiate or see beyond the D1. Subsequently at exploration, right lobe of liver had occupied the position well below the right hemidiaphragm, but duodenum had rotated posteriorly and was compressed between the liver, portal vein, and IVC causing acute angulation and inability to negotiate the tube beyond the first part of duodenum. Because of the abnormal rotated anatomy and comparatively small abdominal cavity, straightening of duodenum was not possible. To relieve obstruction, duodenojejunostomy with feeding jejunostomy was done. Closure of the abdominal wall this time was smooth and without tension. Child was allowed orally on 8th postoperative day. On follow-up, she is doing fine.

## DISCUSSION

When omphalocele defect is larger than 5-6cm, or most of the liver is protruding into the sac, it is termed as giant omphalocele.[1] Treatment options for giant omphalocele include, initial non-operative management and then delayed repair between 6 months to 1 year of age and staged repair using silastic patch over 2-month period.[2,3] Giant omphalocele can be repaired using mesh or tissue expanders. In the present case, right lobe of liver along with gallbladder and part of stomach and duodenum were herniated into the defect, but fascial closure was possible without need of any mesh. Abnormal vascular anatomy of liver due to rotation can cause problems by compression of IVC or hepatic veins while placing the liver back in the abdomen. Importance should also be given to the duodenal anatomy while placing the right lobe of liver back in the abdomen as rotation can later cause duodenal compression and obstruction as happened in our case.

Delayed repair, or resulting ventral hernia repair can be a challenge for surgeons. Infection, inflammation, adhesions, fistula formation, hematoma, seroma, recurrence are the complications known with the use of mesh.[4] We attempted repair without mesh and found it feasible with strict postoperative monitoring. Within a week child’s abdominal cavity accommodated the liver very well.

## Footnotes

**Source of Support:** Nil

**Conflict of Interest:** None declared

